# Phylogenetic and Molecular Evolutionary Insights into Monkeypox Virus Circulation in Shenzhen, China, 2023–2024

**DOI:** 10.3390/v17091214

**Published:** 2025-09-05

**Authors:** Chuan Shi, Xiaochen Zheng, Lei Lei, Jinhui Xiao, Guangqing Yu, Yingdong Li, Zhifeng Ma, Minjie Li, Yanling Zeng, Ziquan Lv, Yixiong Chen, Wei Tan, Qianru Wang

**Affiliations:** 1 School of Medicine, Southern University of Science and Technology, Shenzhen 518055, China; 2 Department of Microbiological Laboratory, Baoan District Center for Disease Control and Prevention, Shenzhen 518101, China; 3 Shenzhen Center for Disease Control and Prevention, Shenzhen 518000, China; 4 State Key Laboratory of Quantitative Synthetic Biology, Shenzhen Institute of Synthetic Biology, Shenzhen Institutes of Advanced Technology, Chinese Academy of Sciences, Shenzhen 518055, China

**Keywords:** monkeypox virus, phylogenetic analysis, single nucleotide polymorphism, APOBEC3

## Abstract

The 2022 global mpox outbreak highlighted the risk of sustained human-to-human transmission of monkeypox virus (MPXV) in non-endemic regions, yet genomic surveillance in Asia, particularly in China, remains limited. This study conducted horizontal genomic surveillance of MPXV in Shenzhen from 2023 to 2024 to characterize the phylogenetic structure, mutational patterns, and adaptive evolution of locally circulating strains. Phylogenetic analysis showed 95.2% of strains belonged to the dominant lineage C.1.1, with 4.8% in lineage E.3, forming three distinct genetic clusters that indicate multiple independent introductions and established local transmission chains. Whole-genome mutational analysis identified 146 single-nucleotide polymorphisms (SNPs), 81.5% of which carried APOBEC3-mediated mutation signatures (TC > TT and GA > AA), reflecting host-driven antiviral editing. Notably, dynamic changes in low-complexity regions (LCRs) were observed, implying potential roles in genome plasticity and adaptive evolution. Functional analysis revealed non-synonymous substitution biases in host-interacting proteins OPG064, OPG145, and OPG210, while replication protein OPG105 remained conserved. Structural modeling identified critical substitutions in OPG002 (S54F), OPG016 (R84K), and OPG036 (R48C) that may enhance immune evasion by modulating TNF-α signaling, NKG2D engagement, and Type I interferon antagonism. These findings illuminate unique MPXV evolutionary dynamics in Shenzhen, emphasizing continuous genomic surveillance for non-endemic outbreak preparedness.

## 1. Introduction

Mpox (formerly known as monkeypox) is a zoonotic disease caused by the monkeypox virus (MPXV), belonging to the *Orthopoxvirus* (OPXV) genus within the *Poxviridae* family [1]. Besides MPXV, the OPXV genus encompasses other human-pathogenic species [2], such as cowpox virus, vaccinia virus, and variola virus (VARV, the causative agent of smallpox) (Figure S1). Morphologically and genomically, MPXV shares core characteristics with its congener VARV; both are brick-shaped enveloped viruses, with MPXV measuring approximately 200–250 nm in diameter and VARV measuring about 200–300 nm, and both harbor linear double-stranded DNA (dsDNA) genomes [3]. The MPXV genome spans approximately 197 kb and encodes over 190 non-overlapping open reading frames (ORFs), a feature conserved in VARV, which encodes a comparable number of ORFs. Both viruses contain a conserved central core region critical for viral replication (e.g., DNA polymerases) and structural protein synthesis (e.g., core membrane proteins supporting virion assembly). This central region is flanked by two variable terminal regions containing inverted terminal repeats (ITRs). Although the ITRs constitute less than 10% of the genome in both viruses, they play pivotal roles in viral–host adaptation and immune evasion, thereby influencing viral pathogenicity and evolutionary dynamics [4]. These characteristics may enhance viral mutation capacity and adaptability to human hosts, particularly in immunocompromised populations, who are more susceptible to severe infection and may serve as potential reservoirs for viral diversification and spread [5]. However, the pathogenesis-related proteins of VARV exhibit significantly higher virulence, whereas those of MPXV are associated with milder clinical manifestations. This difference is consistent with the relatively low case fatality rate (CFR) of MPXV, historically ranging from 3.6% to 10% [6]. Notably, substantial research and epidemiological evidence support the cross-protective effect of smallpox vaccination against mpox [6], a phenomenon attributed to the high sequence homology of conserved proteins between MPXV and VARV.

Mpox is primarily transmitted through direct physical contact with the infectious skin rash, body fluids, or scabs of an infected individual. Indirect contact with contaminated materials, such as fabrics (e.g., clothing, bedding) or surfaces, also constitutes a significant route of transmission. Although the virus can be present in respiratory secretions, transmission via respiratory droplets requires prolonged, close, face-to-face contact and is not considered a common or dominant route of spread in documented outbreaks [7,8]. The severity of mpox is tightly associated with two distinct genetic clades of MPXV: Clade I (formerly Central African clade), characterized by higher virulence and mortality; and Clade II (formerly West African clade, encompassing subclades IIa and IIb), which is linked to milder disease phenotypes. Historically, MPXV was endemic to Central and West Africa, predominantly transmitted via zoonotic spillover from wildlife reservoirs such as rodents and small mammals [2]. However, since May 2022, mpox outbreaks have been reported in numerous non-endemic regions, often without epidemiological links to endemic regions or known animal sources. Between 1 January 2022 and 31 January 2025, over 130,000 confirmed mpox cases, including more than 280 deaths, were reported across 130 countries [9]. This unprecedented global outbreak is characterized by sustained human-to-human transmission, particularly among men who have sex with men (MSM), and a markedly elevated mutation rate, far exceeding the previously estimated OPXV evolutionary rate of 1–2 mutations per year [10,11,12]. Phylogenetic analyses indicate that the MPXV strains driving the 2022 global outbreak share a common ancestor with lineage A.1, linked to the 2017–2019 Nigerian outbreak [13]. A key driver of the observed genetic divergence is thought to be host APOBEC3 cytidine deaminase activity, which induces TC > TT and GA > AA dinucleotide transitions in single-stranded DNA replication intermediates, thereby accelerating viral evolution [11,14].

Research on the 2022 global mpox outbreak has predominantly focused on the evolution of clade IIb B.1 lineage in Europe and North America [15]. In contrast, Asia has experienced a shift from lineage B.1 to C.1.1, with C.1.1 now being the predominant lineage in China (https://nextstrain.org/mpox/clade-IIb, accessed on 29 December 2024) [16,17]. Yet, Asia remains critically underrepresented in global MPXV sequencing efforts, with China contributing fewer than 5% of shared sequences [16]. This underrepresentation severely limits our understanding of lineage turnover, molecular adaptation, and transmission dynamics in the region. Moreover, incomplete transmission chain and limited epidemiological connections prevent accurate viral origin tracing, strategy development for prevention and control, and accurate epidemic prediction.

Shenzhen, a major international port city in Southern China, witnessed rapid local transmission following the detection of its first locally transmitted mpox case in June 2023. Subsequent epidemiological investigations revealed limited transmission links [16,18,19], raising concerns about ongoing cryptic transmission and/or multiple independent introductions. Genomic analyses have shown that MPXV strains from Shenzhen cluster genetically with strains from East Asia (e.g., Japan, South Korea), Western Europe (e.g., Portugal), and other Chinese cities such as Beijing and Hangzhou [16,18]. However, precise transmission routes remain unresolved. This study presents a comprehensive genomic and evolutionary analysis of MPXV strains circulating in Shenzhen from 2023 to 2024. Unlike previous studies limited to single outbreak phases, we adopt a multi-phase surveillance framework encompassing pre-outbreak, peak transmission, and post-outbreak periods. Through integrated phylogenetic reconstruction, mutational profiling, and structural modeling, we elucidate the evolutionary trajectories, lineage-specific genetic markers, and functional impacts of key amino acid mutations, thereby addressing critical gaps in MPXV molecular epidemiology in Shenzhen. Our findings provide actionable insights for outbreak preparedness and surveillance in non-endemic regions through targeted surveillance of high-risk populations, optimization of PCR diagnostics based on mutation signatures, and dynamic risk stratification by integrating mutation burden thresholds.

## 2. Materials and Methods

### 2.1. Clinical Data Collection

This study included 21 laboratory-confirmed mpox cases diagnosed in Shenzhen between June 2023 and August 2024. Diagnosis was based on the presence of compatible clinical symptoms and a positive MPXV PCR result from any anatomical site. Clinical specimens were initially collected for diagnostic purposes. According to the Guidelines for the Diagnosis and Treatment of Monkeypox (2022 edition) issued by the National Health Commission of China, the following sample types were collected from patients with suspected mpox: blood samples (anticoagulated and non-anticoagulated), skin lesion specimens, and nasopharyngeal swabs. Swabs and biopsy specimens were placed in inactivated viral transport medium and subjected to real-time PCR for MPXV DNA detection.

Attending physicians recorded detailed clinical and laboratory information, which was subsequently reported to the Baoan District Center for Disease Control and Prevention. Public health professionals then conducted epidemiological investigations via face-to-face or telephone interviews. A comprehensive epidemiological report was compiled for each case, including information on demographics, symptoms, laboratory findings, prior infection history, travel itinerary within 21 days preceding symptom onset, and documented high-risk exposure behaviors. Detailed clinical data are presented in Table S1.

### 2.2. Genome Sequencing and Assembly

Viral DNA was extracted using the CqEx-DNA/RNA Extraction Kit (Tianlong, Xi’an, China). Samples with the highest viral loads, as determined by cycle threshold values from MPXV-specific real-time PCR, were selected for whole-genome sequencing. Genome amplification was performed following the manufacturer’s instructions using the ATOPlex Multiplex PCR Amplification Kit (MGI, Shenzhen, China). Sequencing libraries were prepared using the MGIEasy Fast PCR-Free FS DNA Library Prep Kit (MGI, Shenzhen, China), quantified with a Qubit 4.0 Fluorometer (Invitrogen, Carlsbad, CA, USA), and normalized. Circularization was conducted using the DNBSEQ OneStep DNB Make Reagent Kit (MGI, Shenzhen, China) to generate DNA nanoballs (DNBs), which were then sequenced on the DNBSEQ-G99 platform.

Raw sequencing reads were quality-filtered using SOAPnuke v1.5.2 (https://github.com/BGI-flexlab/SOAPnuke, accessed on 6 December 2024) to remove adapter sequences and low-quality reads [20]. Host-derived sequences were removed by aligning filtered reads to the human reference genome using BWA v0.7.12 (https://github.com/lh3/bwa, accessed on 6 December 2024) [21]. The remaining MPXV-specific reads were mapped to the reference genome (GenBank accession: NC_063383.1) using MAQ v0.7.1 (https://github.com/maqsoftware, accessed on 6 December 2024) for reference-based assembly [22]. The 21 assembled MPXV genomes have been submitted to the NCBI database under accession numbers from PV785646.1 to PV785666.1. Sequencing details are provided in Table S2.

### 2.3. Phylogenetic and Evolutionary Analysis

A total of 191 high-quality MPXV genome sequences, including temporal and geographic metadata, were retrieved from NCBI database (https://www.ncbi.nlm.nih.gov, updated as of 31 March 2025). Additionally, 29 MPXV genomes from the China National Center for Bioinformation (CNCB) database (https://ngdc.cncb.ac.cn/gwh/poxvirus, accessed on 31 March 2025; Accession: CRA015345; BioProject: PRJCA024271) were included. Detailed information on all sequences analyzed is provided in Table S3.

Evolutionary lineage assignments were performed using the NextClade online platform (https://nextstrain.org, accessed on 10 April 2025). Multiple sequence alignment was conducted using MAFFT v7.475 (https://github.com/GSLBiotech/mafft, accessed on 10 April 2025) [23]. Phylogenetic relationships were inferred using the maximum-likelihood method implemented in RAxML v8.2.12 (https://github.com/amkozlov/raxml-ng, accessed on 10 April 2025) within Geneious Prime 2024.0, employing general time-reversible gamma substitution model [24]. Node support was evaluated with 1000 ultrafast bootstrap replicates. Clades with bootstrap support values ≥ 70 were considered statistically significant. The resulting phylogenetic tree was visualized using FigTree v1.4.2 (http://tree.bio.ed.ac.uk/software/figtree/, accessed on 20 April 2025).

### 2.4. Sequence Diversity and Mutation Profiling

Tandem repeat sequences (TRSs) were identified using Tandem Repeats Finder v4.09 (https://github.com/Benson-Genomics-Lab/TRF, accessed on 12 April 2025) [25] with the following parameters: matching weight 2, mismatching penalty 7, indel penalty 7, and minimum alignment score 50. All candidate TRSs were manually curated to ensure positional accuracy and repeat unit consistency. Nucleotide and amino acid mutations in the 21 MPXV genomes were identified relative to the clade IIb reference genome (NC_063383.1). Mutation sites were extracted and visualized using the Snipit tool v1.6 (https://github.com/aineniamh/snipit, accessed on 12 April 2025). Complete variant information is listed in Table S4.

### 2.5. Prediction of Impacts of Mutations on Protein Structure and Function

Three-dimensional structural models of proteins encoded by OPG002, OPG016, and OPG047 were predicted using the AlphaFold 3 server (https://github.com/google-deepmind/alphafold3, accessed on 12 June 2025) [26]. Only models with a predicted template modeling score (pTM) > 0.6 were retained for analysis. Mutation sites were interpreted structurally only if their local predicted local distance difference test (pLDDT) scores exceeded 70. Structural visualization and mutation mapping were performed using PyMOL v3.1.6.1 (http://www.pymol.org/, accessed on 12 June 2025). Protein stability changes were quantified by calculating differences in the Gibbs free energy (ΔΔG) between wild-type and mutant variants using I-Mutant 2.0 (https://folding.biofold.org/i-mutant/i-mutant2.0, accessed on 12 June 2025), with negative ΔΔG values indicating stabilizing effects.

### 2.6. Statistical Analysis

Descriptive statistics were used to summarize patient characteristics. Continuous variables were reported as mean, median, and interquartile range. Categorical variables were expressed as frequencies and percentages.

## 3. Results

### 3.1. Demographic and Clinical Characteristics of Confirmed Mpox Cases

This study analyzed 21 laboratory-confirmed mpox cases in Shenzhen (June 2023–August 2024; Table S1). The cohort consisted exclusively of men (median age, 29.0 years; interquartile range, 26.5–32.5), all identifying as MSM individuals with no contact tracing-derived epidemiological connections. Travel history analysis revealed one imported case (4.8%; recent international travel to Thailand within 21 days prior to symptom onset) and eight domestic mobility cases (38.1%). Behavioral risk assessment documented high-risk sexual exposures in 14 cases (66.7%; multiple/anonymous sexual partners), including four (19.0%) participating in high-risk sexual gatherings. Clinical manifestations comprised skin lesions (100%), fever (85.7%), and lymphadenopathy (33.3%). Co-infections were prevalent, including HIV infection (*n* = 10, 47.6%; all on antiretroviral therapy) and other sexually transmitted infections (STIs): syphilis (*n* = 6, 28.6%) and HSV-1 infection (*n* = 1, 4.8%).

### 3.2. Phylogenetic Characterization of MPXV Strains Circulating in Shenzhen

**Figure 1:**
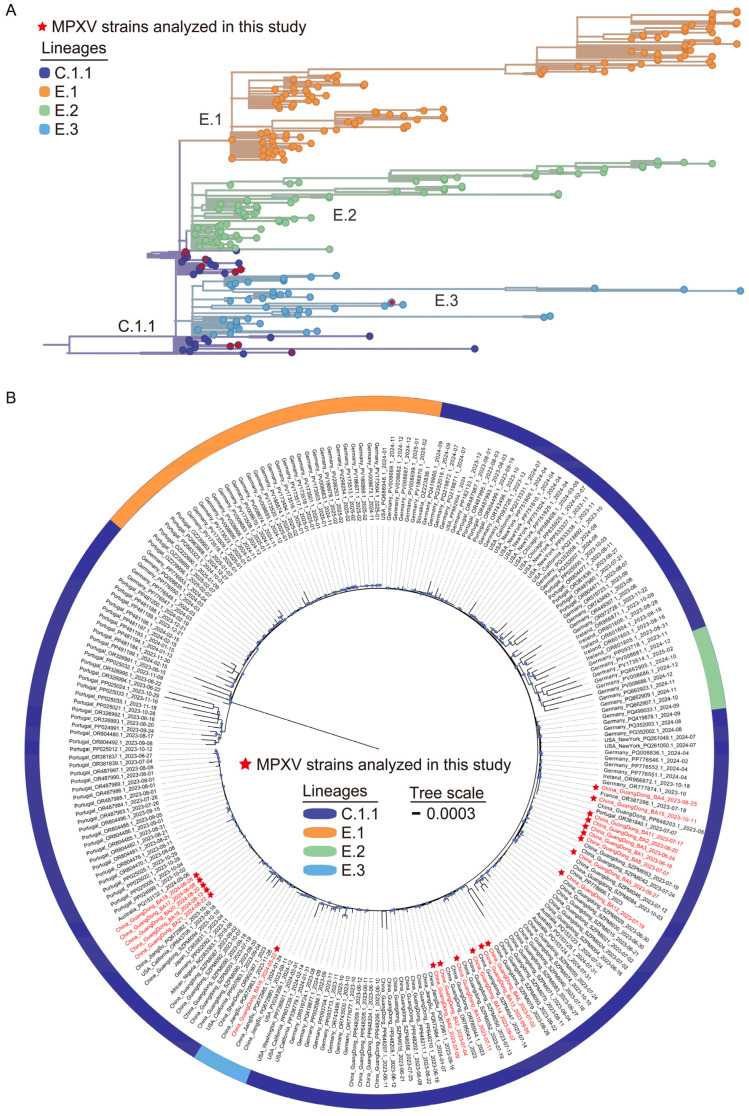
Phylogenomic analysis of local MPXV genome sequences from Shenzhen during 2023–2024. (**A**) Phylogenetic lineage tracing of 21 Shenzhen MPXV genomes. Branch labels in different colors represent distinct lineages. The Shenzhen MPXV genomes are marked with red stars. (**B**) Maximum-likelihood phylogenomic tree incorporating the 21 Shenzhen MPXV genomes, alongside globally representative lineage C.1.1 and descendant lineage genomes retrieved from the NCBI (191) and CNCB (29) database. Red-labeled branches represent the Shenzhen MPXV genomes. Blue dots on branches indicate bootstrap support values ≥ 70.

The 2022 global mpox outbreak was predominantly associated with clade IIb lineage B.1 [19], which subsequently underwent substantial diversification into multiple distinct lineages and sub-lineages through cumulative nucleotide substitutions and recombination events across different geographic regions. The lineage B.1.3 evolved into lineages E.1–E.3 via the C.1.1, while lineage B.1.20 further diversified into lineages F.1–F.6 (https://nextstrain.org/mpox/clade-IIb, accessed on 31 March 2025). Among the 21 MPXV strains sequenced from Shenzhen cases, phylogenetic classification showed twenty strains (95.2%) clustered within lineage C.1.1, with a single strain (4.8%) assigned to lineage E.3 (Figure 1A).

To characterize the genomic and molecular evolution of MPXV strains circulating in Shenzhen, a maximum-likelihood phylogenomic tree was constructed by integrating the 21 Shenzhen MPXV genomes with 220 globally representative lineage C.1.1 and descendant lineage genomes from NCBI and CNCB databases (Figure 1B). This analysis revealed three distinct genetic clusters among Shenzhen strains, suggesting that the Shenzhen mpox outbreak likely resulted from several independent introduction events followed by sustained local dissemination. The majority of Shenzhen strains (*n* = 20) clustered closely with MPXV strains from other cities within Guangdong Province and global strains from Australia and European (Portugal, France, Germany). In contrast, the E.3 strain of Shenzhen (China_GuangDong_BA16_2024-05-22) clustered with strains from Jiangsu Province of China and the United States. These findings demonstrate the complexity of MPXV circulation in Shenzhen, characterized by multiple cross-border introductions (international and interprovincial) and silent local transmission chains, warranting further epidemiological investigation.

### 3.3. Molecular Evolution and Mutation Signatures of MPXV Strains in Shenzhen

**Figure 2:**
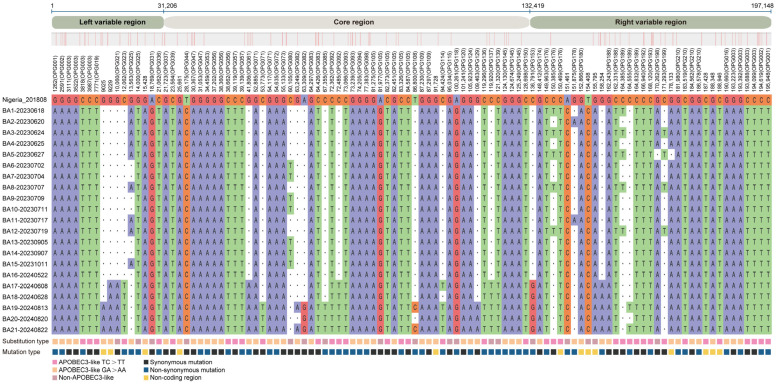
Nucleotide diversity analysis of Shenzhen MPXV genome sequences. Comparative analysis of 21 Shenzhen MPXV strains against the reference IIb B.1 strain (NC_063383.1) revealed 146 distinct SNPs. The private mutations (those unique to individual strains) were not displayed. • represents at a given position any type of nucleotide. The color-coded bar at the bottom of the figure denotes the types of nucleotide substitutions. Complete SNP data are presented in Table S4.

**Figure 3:**
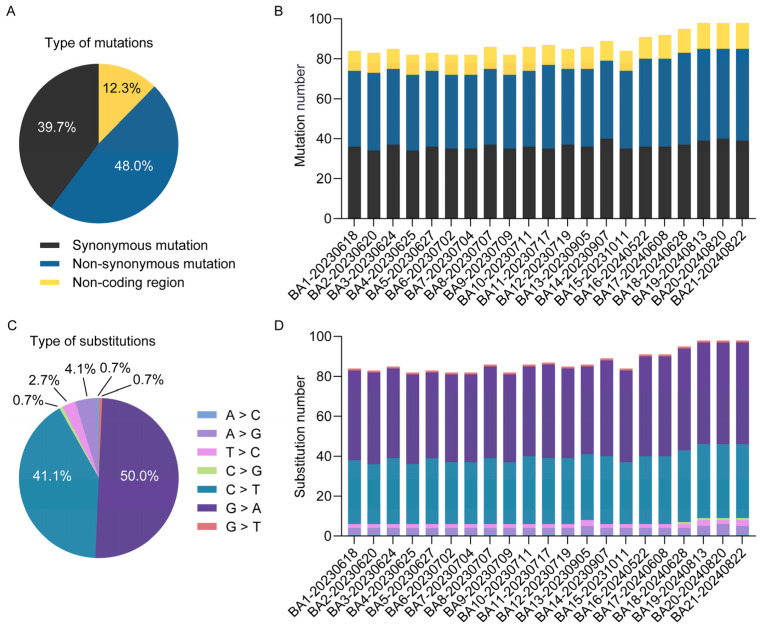
Molecular evolution characteristics of Shenzhen MPXV genomes. The proportions (**A**) and genomic distribution (**B**) of mutation types, along with the proportions (**C**) and distribution (**D**) of nucleotide substitution types, are presented for 21 Shenzhen MPXV genomes.

To elucidate the microevolutionary dynamics of circulating MPXV strains, we conducted whole-genome sequence alignment of all 21 Shenzhen isolates against the 2018 Nigerian reference genome (NC_063383.1) (Figure 2 and Table S4). Our analysis revealed a total of 146 SNPs (Figure 3A), consisting of 70 non-synonymous mutations (48.0%), 58 synonymous mutations (39.7%), and 18 intergenic mutations (12.3%). Genomic distribution analysis demonstrated significant regional variation in mutation density, with the left variable region (segment 1–31,205, OPG001–OPG048) containing 30 mutations (20.5%), the right variable region (segment 132,420–199,148, OPG152–OPG210) harboring 51 mutations (34.9%), and the conserved central region (segment 31,206–132,419, OPG049–OPG151) accumulating 64 mutations (43.8%). The mutation spectrum was further classified based on prevalence frequency: 79 mutations (54.1%) were conserved across all isolates (shared), 25 (17.1%) appeared in multiple but not all strains (partially shared), and 42 (28.8%) were strain-specific (private). Temporal analysis revealed accelerated genomic diversification, with late-phase isolates (2024 collection; strains NO. 16–21; 95.3 mutations per strain) displaying 1.2-fold higher mutation burden compared to early-phase strains (2023 collection; strains NO. 1–15; 79.1 mutations per strain) (Figure 3B,D). Notably, we identified several novel mutations emerging exclusively in 2024 isolates (Figure 2): C21062T (OPG023), G41806A (OPG061), C119920T (OPG137), and C136791G (OPG153). These temporally restricted mutations may represent potential adaptive changes associated with prolonged human-to-human transmission.

### 3.4. APOBEC3-Mediated Mutations Drive Selective Evolution of MPXV

**Figure 4:**
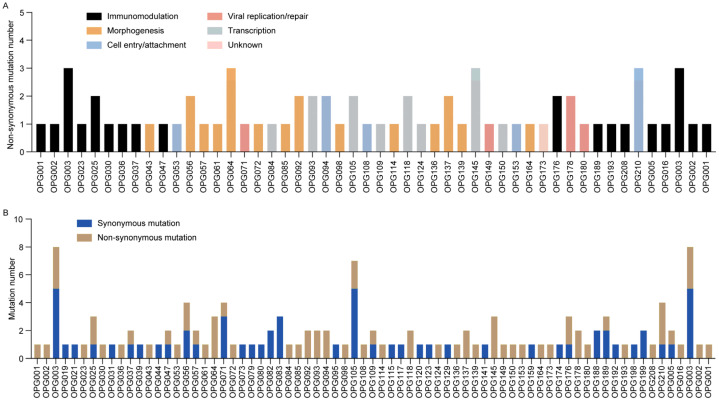
Distribution and functional classification of mutations in Shenzhen MPXV genomes. (**A**) Bar chart illustrating non-synonymous mutation counts per OPG, color-coded by protein functional category. (**B**) Stacked bar chart displaying mutation type frequencies across OPGs in 21 MPXV genomes.

Systematic analysis of nucleotide substitution patterns across 21 MPXV genomes revealed that G > A (50.0%, 73/146) and C > T (41.1%, 60/146) transitions were the dominant mutation types (Figure 3C). Notably, GA > AA substitutions accounted for 44.5% of all mutations, while TC > TT substitutions represented 37.0% (54/146) (Table S4). Together, these transitions constituted 81.5% of all observed substitutions. This mutation pattern closely aligns with the known mutational bias associated with APOBEC3 enzymatic activity, which has been implicated in driving MPXV evolution [27]. Protein-specific evolutionary analysis demonstrated distinct selection pressures: OPG003 (ankyrin repeat protein) showed high rates of both non-synonymous and synonymous substitutions, while OPG064 (Iev morphogenesis), OPG145 (DNA helicase), and OPG210 (B22R family protein) exhibited elevated non-synonymous rates (Figure 4A), contrasting with OPG105 (RNA polymerase rpo147) which maintained high non-synonymous constraint (Figure 4B). Functional categorization revealed immune/virulence-associated proteins accumulated predominantly adaptive non-synonymous mutations, whereas structural/replication proteins were characterized by synonymous mutations, demonstrating differential evolutionary pressures across viral protein functional classes during MPXV adaptation to human hosts.

### 3.5. Predicted Structural and Functional Impact of Key Amino Acid Substitutions

**Figure 5:**
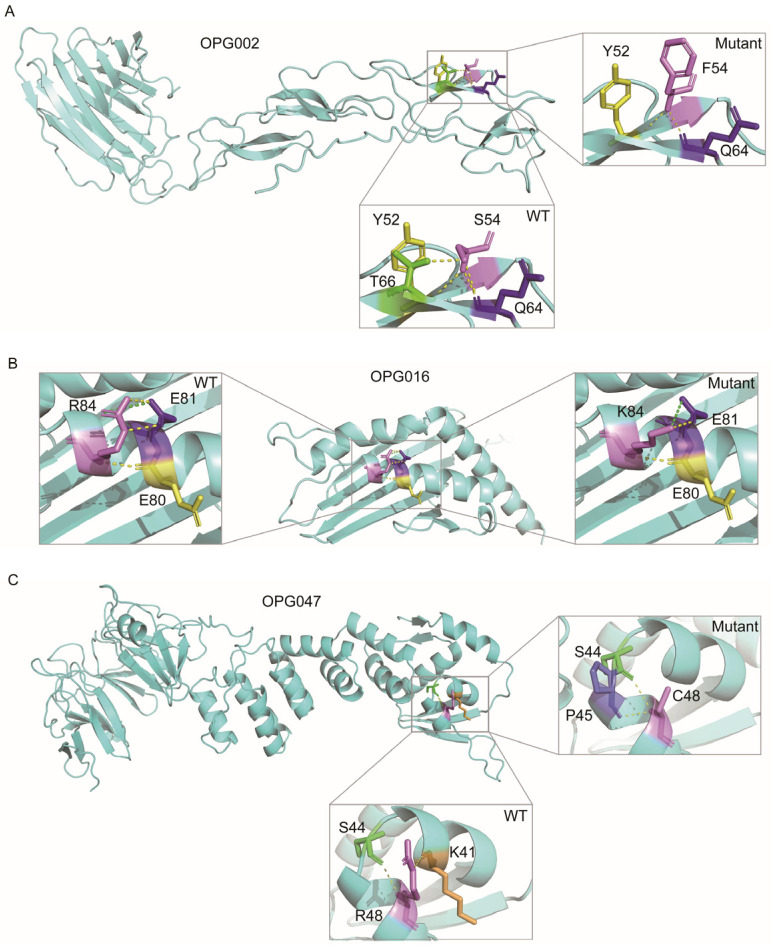
Structural comparison of MPXV proteins harboring critical mutations. Conformational differences between wild-type (WT) and mutant variants of OPG002 (**A**), OPG016 (**B**), and OPG047 (**C**) proteins are presented, with magnified views highlighting mutated residues. Hydrogen bonds and salt bridges are denoted by yellow and green dashed lines, respectively.

To elucidate the functional roles of MPXV proteins, a systematic functional annotation was conducted (Table S5). Three immunomodulatory proteins were prioritized for detailed investigation: OPG002 (TNF-α receptor homolog Crm-B), OPG016 (Brix domain protein), and OPG047 (Kelch-like protein) (Figure 5 and Table 1). These were selected based on their evolutionary signatures and well-established roles in immune evasion. Moreover, their variants exhibit significant residue property divergence as well as substantial ΔΔG changes. In OPG002, the S54F substitution within a β-sheet domain introduced steric hindrance due to the larger aromatic ring of phenylalanine, disrupting hydrogen bonding and decreasing β-sheet stability. This mutation also affects TNF-α binding domain architecture, potentially impairing recognition and binding affinity of TNF-α. In OPG016, the R84K mutation, situated in an α-helix domain, exhibits a markedly negative ΔΔG value (−2.7 kcal/mol), indicating enhanced conformational stability. Although lysine disrupts hydrogen bonds, the formation of an optimized salt bridge compensates for it, enhancing structural stability. This mutation occurs within the MHC class I-like antigen recognition domain, suggesting a potential mechanism for viral evasion of NK cell surveillance by modulating interactions with activating receptors. Lastly, the R48C mutation in OPG047 disrupts an interhelical hydrogen bond in the BTB domain’s α-helix while introducing a new hydrogen bond in the helix linker region. The substitution of positively charged arginine with hydrophobic cysteine alters the local electrostatic microenvironment and reduces polarity, potentially impairing substrate-binding specificity, a critical function of the BTB domain in ubiquitination pathways. These structural perturbations collectively demonstrate how targeted amino acid substitutions in viral immune modulators may enhance host adaptation through both stability optimization and functional domain modification.

### 3.6. Tandem Repeat Variability in Low-Complexity Regions Drives Evolutionary Adaptation of MPXV

Comprehensive genomic analysis of short tandem repeats (STRs) and homopolymers identified two polymorphic low-complexity regions (LCRs, including STRs and homopolymers) exhibiting structural variations relative to the reference strain (NC_063383.1) (Figure S2). LCR1 (position 133,164), containing a [CAATCTTTCT]n motif, displayed a fixed deletion in all 21 Shenzhen strains (*n* = 1 repeat unit) compared to the reference genome (*n* = 2), suggesting this locus underwent selective constraint during viral adaptation. In contrast, LCR2 (position 163,190) featured a dynamic [TAAC]n repeat, with 90.5% (19/21) of strains maintaining the reference configuration (*n* = 6) while 9.5% (2/21) exhibited expansion to seven units. This length polymorphism in LCR2 indicates ongoing adaptive evolution, potentially mediating genomic plasticity through slipped-strand mispairing or recombination-based mechanisms.

## 4. Discussion

This study presents a comprehensive characterization of clinical manifestations, epidemiological patterns, and virological properties among 21 laboratory-confirmed mpox cases in Shenzhen, China (June 2023–August 2024). Our genomic epidemiology findings delineate distinct evolutionary trajectories and transmission networks of circulating MPXV strains in Shenzhen, offering valuable insights for refining public health interventions in non-endemic regions.

The demographic and transmission characteristics observed in Shenzhen mpox cases exhibit concordance with global patterns from the 2022 multinational outbreak [8]. While dermatological evaluations confirmed universal cutaneous manifestations (100%), accompanied by frequent systemic symptoms including fever (85.7%) and lymphadenopathy (33.3%), potential diagnostic variability necessitates implementation of standardized clinical assessment protocols. Epidemiological investigations were constrained by both behavioral factors (concealed mobility within MSM populations) [19] and limitations of passive surveillance, including probable underreporting and undetected asymptomatic infections. The substantial HIV-1 co-infection prevalence (47.6%) underscores critical intersections between mpox and HIV/AIDS surveillance systems, advocating for integrating mpox surveillance into existing HIV/AIDS management frameworks to enhance early detection and optimize resource allocation.

From 2022 to 2024, the epicenter of the mpox outbreak transitioned from Europe and the Americas to the Asia–Pacific region, notably affecting South Korea, Japan, and China. China’s delayed outbreak onset, combined with unique sociocultural determinants, complicates transmission modeling predictions. Current genomic datasets exhibit significant geographical bias, with overrepresentation of South Korean and Japanese strains [3], potentially underestimating the diversity and distribution of MPXV lineages across other Asian regions. Previous studies have primarily relied on early-phase data, lacking systematic long-term dynamic tracking. Our genomic surveillance (June 2023–August 2024) addresses this gap, identifying lineage C.1.1 as predominant (95.2%), with minor circulation of lineage E.3 (4.8%). Phylogenetic analysis demonstrated that Shenzhen’s predominant C.1.1 strains clustered closely with those from other cities within Guangdong province (China), Australia, and Europe (Portugal, France, Germany), while the minor E.3 lineage exhibited genetic similarity to strains from Jiangsu province (China) and the United States. These findings demonstrate a dual transmission pattern in Shenzhen, characterized by both localized community transmission and cross-regional importation events, highlighting the need for targeted epidemiological investigations to elucidate transmission dynamics.

Contemporary models of MPXV molecular evolution identify three primary mechanisms: mutation accumulation, genomic recombination, and codon usage bias. A significantly higher prevalence of APOBEC3-mediated mutations was observed compared to that reported in early-phase pandemic studies [11,28]. Specifically, 81.5% of SNPs exhibit APOBEC3-type mutation signatures, which is consistent with findings from Europe, North America, and other regions [14,29,30], suggesting convergent evolution under human adaptive pressures. Temporal analysis demonstrated a progressive accumulation of non-APOBEC3 mutations. Notably, the proportion of non-APOBEC3-type mutations was substantially higher among partially shared (21.4%) and private mutations (32%) compared to shared mutations (12.6%), indicating a potential threshold effect. This pattern suggests an evolutionary equilibrium where moderate APOBEC3-mediated mutagenesis provides selective advantages, while excessive mutation burden may compromise viral fitness and transmission efficiency.

The increased mutation burden in later-phase strains may reflect adaptive evolution of MPXV under prolonged human-to-human transmission, which aligns with global evolutionary trends of MPXV [11,14]. In this study, the novel mutations identified in later-phase strains, such as C21062T (OPG023), C119920T (OPG137), and C136791G (OPG153), exhibit significant implications for immune evasion, viral fitness, and transmissibility. The homologous protein D7L of OPG023, an ankyrin-like protein, is likely involved in host range determination [28]. OPG137 encodes a virion membrane assembly protein that is involved in the formation of the crescent membrane and immature virion [31]. OPG153 is involved in virus attachment and egress, and correlates with viral replication as well as particle morphology [32].

LCRs also serve as critical determinants of MPXV host adaptation and virulence modulation [33]. These regions demonstrate significantly higher variability than SNPs and display non-random genomic distribution patterns across defined coding regions [32]. Most notably, LCR1 (segment 133,164–133,183) is located within an evolutionarily conserved genomic region (locus 130,000–138,000), which has been functionally associated with OPXV virulence factors and transmission mechanisms [32], indicating its potential evolutionary significance in OPXV pathogenesis.

Molecular evolutionary analysis revealed distinct evolutionary signatures in MPXV proteins. Viral–host interaction proteins, such as OPG064 (Iev morphogenesis protein) and OPG210 (B22R family protein), display elevated non-synonymous mutation rates, suggesting either relaxed purifying selection or adaptive evolution under selective pressures. These amino acid substitutions may optimize virus–host interface dynamics to enhance immune evasion or infection efficiency under selective pressures. Conversely, OPG105 (RNA polymerase subunit) showed predominantly synonymous mutations, reflecting evolutionary stability on core replication machinery [28]. This disparity in adaptive diversification among host-interacting proteins versus conserved evolution in replication-related proteins reflects a strategic balance between viral plasticity and replication stability. Notably, while hotspot mutations identified in Shenzhen MPXV strains were consistently detected in global strains [11,29,30], Shenzhen strains exhibited higher mutation accumulation in immunomodulatory proteins (e.g., OPG003, OPG210) [28,30,34], suggesting accelerated host adaptation in Asian strains.

Structural modeling of immunomodulatory proteins identified functionally consequential substitutions: OPG002-S54F disrupts TNF-α binding pocket topology [35,36]; OPG016-R84K enhances NKG2D receptor engagement via optimized electrostatic interactions [34,37]; OPG047 mutations remodel BTB/Kelch domain interfaces, potentially altering interferon antagonism [33,38,39]. These findings reveal a convergent evolutionary strategy targeting TNF-α signaling, NKG2D receptor engagement, and Type I interferon pathways, collectively suppressing NK cell cytotoxicity through complementary mechanisms.

This study has several limitations. First, the relatively small sample size (*n* = 21) may constrain the statistical power to detect rare mutations and subtle transmission dynamics. Future studies should enroll larger cohorts spanning multiple epidemic waves to address this limitation. Second, while structural predictions were generated using computational modeling, these in silico analyses require complementary experimental validation (e.g., in vitro functional assays, structural determination via cryo-EM) to enhance the accuracy of functional interpretations.

## Figures and Tables

**Table 1:** Structural and functional impacts of critical amino acid substitutions.

Gene	Protein	Amino Acid Mutation	Structural Domain	ΔΔG (kcal/mol)	Predicted Molecular Effects	Predicted Functional Implications
OPG002	Crm-B secreted TNF-alpha-receptor-like protein	OPG002: S54F	TNF-α binding domain	−0.50	Steric hindrance by aromatic ring	Impaired TNF-α binding affinity
					Disrupted H-bond network	
OPG016	Brix domain protein	OPG016: R84K	MHC-I-like domain	−2.70	Optimized salt bridge formation	Enhanced NKG2D evasion
OPG047	Kelch-like protein	OPG047: R48C	BTB domain	−1.38	New H-bond in linker region	Altered interferon antagonism
					Altered electrostatic potential	

## Data Availability

The datasets generated in this study are available in public repositories, with repository names and accession numbers provided in the article and Supplementary Tables.

## Supplementary Materials

The following supporting information can be downloaded at: https://www.mdpi.com/article/10.3390/v17091214/s1, Figure S1: Maximum-likelihood phylogenetic tree constructed from an alignment of core genes of orthopoxviruses; Figure S2: Sequence diversity in low-complexity regions of Shenzhen MPXV genomes; Table S1: Clinical and epidemiological details of 21 cases infected with MPXV in Shenzhen; Table S2: Whole-genome sequencing and genomic characteristics of 21 MPXV strains from Shenzhen, China; Table S3: Complete list of MPXV genome sequences (*n* = 220) retrieved from the NCBI and CNCB databases (as of 31 March 2025); Table S4: SNPs distinguishing 21 Shenzhen MPXV strains from the reference IIb B.1 genome (GenBank: NC_063383.1); Table S5: MPXV proteins and their characteristics (GenBank: NC_063383.1).
