# Supplementary figures and images for: Phylogenetic and Molecular Evolutionary Insights into Monkeypox Virus Circulation in Shenzhen, China, 2023–2024
Source: Viruses. 2025 Sep 5;17(9):1214. doi: 10.3390/v17091214 (PMC12474256; doi:10.3390/v17091214)

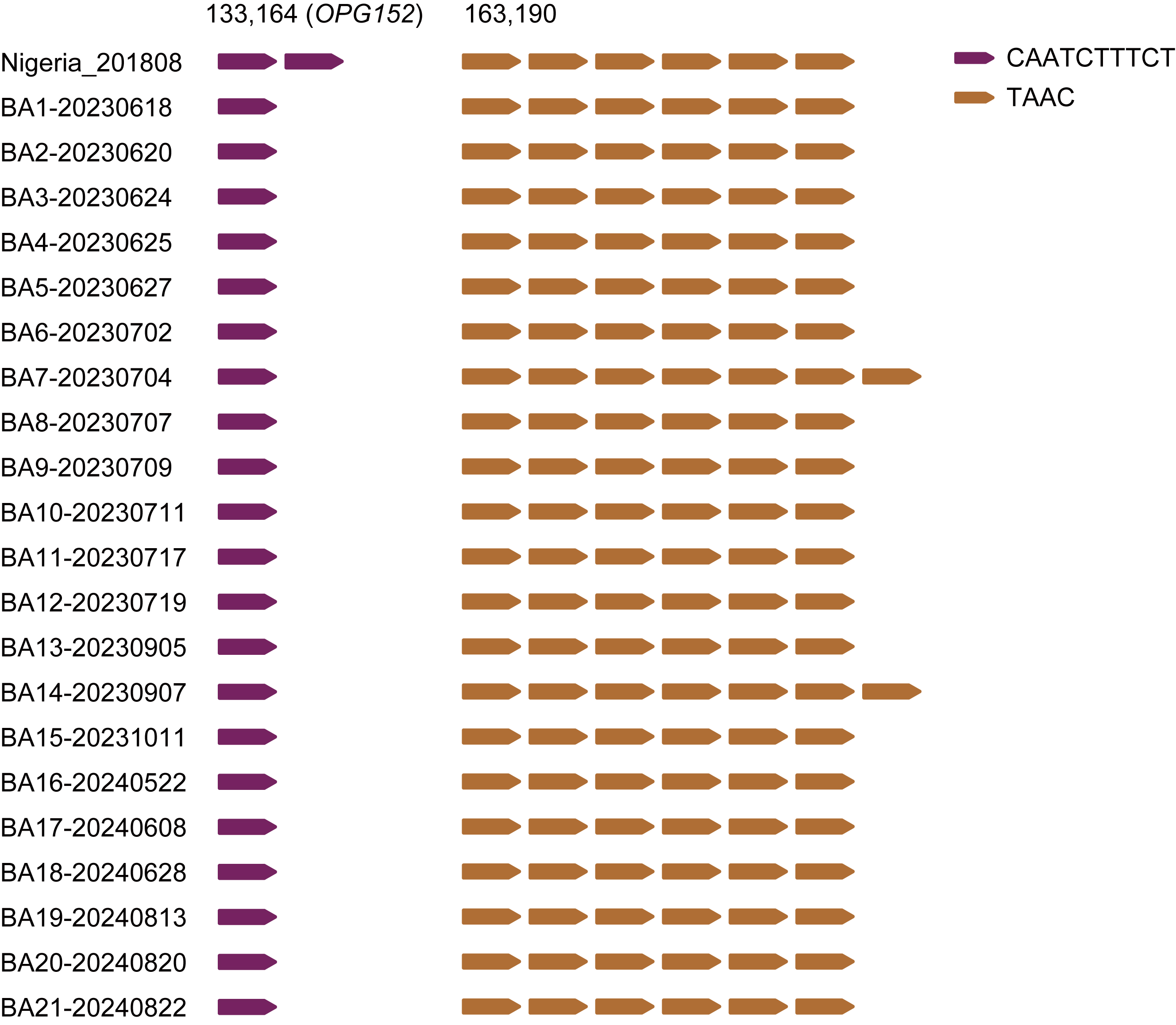

Supplement: Supplementary file 1 [file viruses-17-01214-s001.zip › Figure S2.tif]

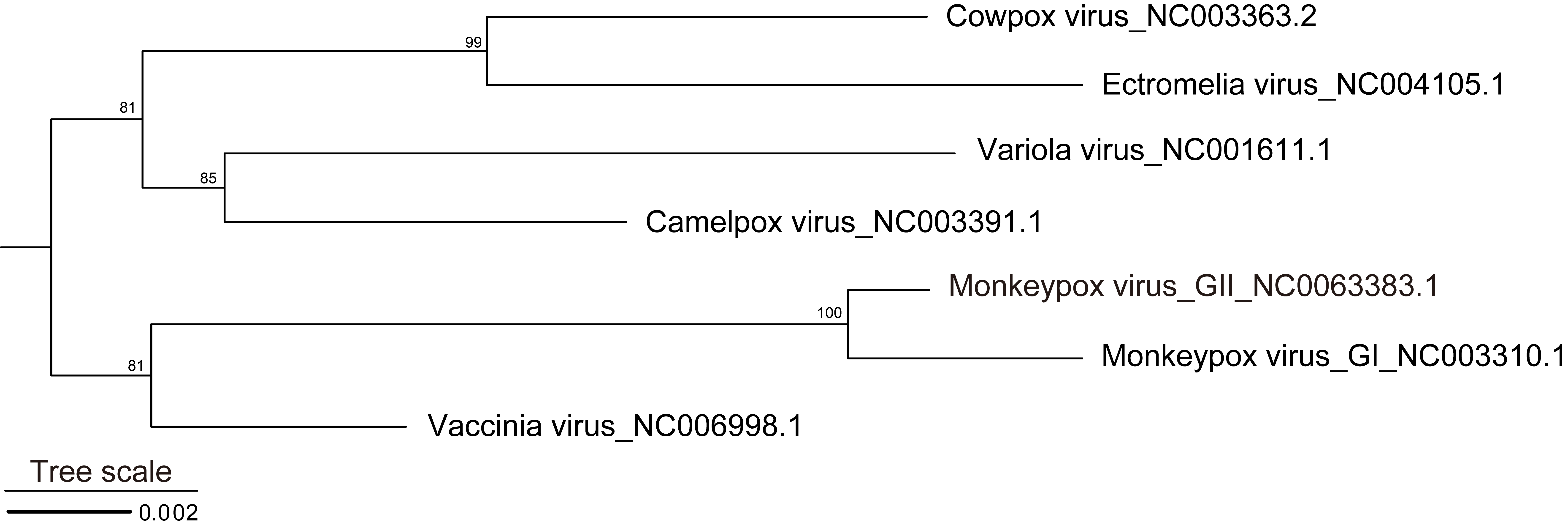

Supplement: Supplementary file 1 [file viruses-17-01214-s001.zip › Figure S1.tif]
